# ICAD Deficiency in Human Colon Cancer and Predisposition to Colon Tumorigenesis: Linkage to Apoptosis Resistance and Genomic Instability

**DOI:** 10.1371/journal.pone.0057871

**Published:** 2013-02-22

**Authors:** Youssef Errami, Hassan Brim, Karine Oumouna-Benachour, Mustapha Oumouna, Amarjit S. Naura, Hogyoung Kim, Jihang Ju, Christian J. Davis, Jong G. Kim, Hassan Ashktorab, Kenneth Fallon, Ming Xu, Jianhua Zhang, Luis Del Valle, A. Hamid Boulares

**Affiliations:** 1 The Stanley Scott Cancer Center, Louisiana State University Health Sciences Center, New Orleans, Louisiana, United States of America; 2 Department of Pathology and Cancer Center, Howard University, Washington, D.C., United States of America; 3 Department of Pathology, University of Alabama, Birmingham, Alabama, United States of America; 4 Department of Anesthesia and Critical Care, the University of Chicago, Chicago, Illinois, United States of America; Ohio State University Comprehensive Cancer Center, United States of America

## Abstract

We previously showed that DNA fragmentation factor, which comprises a caspase-3-activated DNase (CAD) and its inhibitor (ICAD), may influence the rate of cell death by generating PARP-1-activating DNA breaks. Here we tested the hypothesis that ICAD-deficient colon epithelial cells exhibiting resistance to death stimuli may accumulate additional genetic modifications, leading to a tumorigenic phenotype. We show that ICAD deficiency may be associated with colon malignancy in humans. Indeed, an examination of ICAD expression using immunohistochemistry in an array of both colon cancer and normal tissues revealed that ICAD expression levels were severely compromised in the cancerous tissues. Upon DNA damage caused by a low dose of irradiation, ICAD cells acquire a tumorigenic phenotype. Colon epithelial cells derived from ICAD mice showed a significant resistance to death induced by the colon carcinogen dimethylhydrazine in vitro and in mice. Such resistance was associated with a decrease in PARP-1 activation. In an animal model of dimethylhydrazine-induced colon tumorigenesis, ICAD^−/−^ mice developed significantly higher numbers of tumors with markedly larger sizes than the wild-type counterparts. Interestingly, the phenotype of the ICAD^−/−^ mice was not associated with a significant increase in the precancerous aberrant crypt foci suggesting a potential link to tumor progression rather than initiation. More importantly, ICAD deficiency was associated with severe genomic instability as assessed by array comparative genomic hybridization. Such genomic instability consisted most prominently of amplifications but with sizable deletions as compared to the wild-type counterparts affecting several cancer-related genes including *RAF-1*, *GSN*, *LMO3*, and *Fzd6* independently of *p53*. Altogether, our results present a viable case for the involvement of ICAD deficiency in colon carcinogenesis and show that apoptosis and genomic instability may comprise the means by which such deficiency may contribute to the process of increasing susceptibility to carcinogen-induced tumorigenesis.

## Introduction

Colon carcinogenesis is a multistep process that requires many years to develop and is associated with several recently characterized genetic alterations [1], [2], [3]. The occurrence of inactivating mutations in both alleles of the *adenomatous polyposis coli* gene (*APC*) [2], a tumor suppressor gene, is considered to be one of the initial events in colorectal carcinogenesis. Mutation and consequent dysregulation of the *K-RAS* proto-oncogene are also thought to constitute an early contributing factor to colon carcinogenesis [4]. The turnover of epithelial cells is relatively rapid and genetic alterations that render these cells resistant to apoptosis or more proliferative are critical initiating events in colon tumorigenesis [4]. The subsequent accumulation of mutant epithelial cells contributes to formation of structures known as aberrant crypt foci (ACF) [5].

Fragmentation of DNA is thought to be an important step in disposal of the genome of apoptotic cells. Concomitant with cleavage of nuclear lamin, DNA is first degraded into large fragments of 50 to 300 kb, which are then processed into internucleosomal repeats [6], [7]. DNA fragmentation factor (DFF) has been suggested to contribute to this process [8]. DFF is composed of two subunits, a 40-kDa caspase-activated DNase (CAD) or DFF40 and its 45-kDa inhibitor ICAD (DFF45) [8], [9], [10]. The endonuclease activity of this enzyme, which is intrinsic to CAD, is induced on cleavage of ICAD by caspase-3. ICAD functions both as an inhibitor of CAD activity and as a chaperone for this subunit [10], [11], [12]. Indeed, ICAD is required for CAD expression and the abundance of the endonuclease in ICAD^−/−^ cells is greatly reduced compared with that in wild type cells [8], [9], [10], [13]. We and others [13], [14], [15], [16], [17] reported that a variety of cell types, including immune cells, fibroblasts, and cortical neurons derived from ICAD^−/−^ mice, exhibit resistance to apoptosis induced by various stimuli. We also demonstrated the importance of DFF in the fragmentation of DNA into 50-kb pieces during apoptosis [13], [14], [15], [16], [17]. The generation of these DNA strand breaks results in the early and transient activation of PARP-1. PARP-1-mediated poly(ADP-ribosyl)ation results in a marked decrease in the intracellular concentrations of NAD and ATP, creating a cellular energy shortage that is thought to contribute to acceleration of the death process [13], [16], [18], [19]. We showed that failure of ICAD^−/−^ fibroblasts to generate 50-kb DNA fragments in response to TNF-α protected the cells from excessive activation of PARP-1 and thereby prevented depletion of intracellular NAD [13], [16]. The delay in PARP-1 activation observed in these cells correlated with delays both in caspase-3 activation and in pro-apoptotic mitochondrial events, including the loss of mitochondrial membrane potential and the release of cytochrome c [13], [16]. Based on these various observations, we proposed that the generation of 50-kb DNA fragments by DFF is not merely a passive step in DNA degradation but rather that it contributes, together with PARP-1, mitochondria, and caspase-3, to an amplification phase of apoptosis [13], [16].

ICAD expression and mutations in the *ICAD* gene were recently associated with a number of human malignancies as well as with an animal model of skin cancer [20], [21]. In addition, Konishi et al. reported that ICAD mRNA expression was lower in esophageal squamous cell carcinoma tumors with higher pathologic grade along with lymph node metastasis [20]. Conversely, ICAD protein expression was reported to be frequently upregulated in ovarian serous carcinomas and may serve as a marker of aggressive behavior with prognostic value [22]. Given the importance of apoptosis in maintenance of colonic tissue and its dysregulation in colon carcinogenesis, we intended in the present study to examine whether the potential association between involvement of ICAD in apoptosis and genomic stability may be critical in preventing colon tumorigenesis. Additionally, we sought to determine whether the failure of colonic epithelial cells to express ICAD rendered them susceptible to accumulate genetic aberrations in addition to a potential ability to resist induction of apoptosis, increasing the susceptibility of these animals to carcinogen (dimethylhydrazine; DMH)-induced colon tumorigenesis. For these studies, we used an integrative approach combing an array of human colon cancer and normal tissues, a newly developed method for isolating primary colon epithelial cells, and a well-established DMH-induced mouse model of colon carcinogenesis.

## Materials and Methods

### Ethics Statement

The current study was conducted in strict accordance with the recommendations in the Guide for the Care and Use of Laboratory Animals of the National Institutes of Health. Maintenance (IACUC protocol: BC0015) and experimental protocol (IACUC protocol: 2227) were approved by the LSUHSC Animal Care and Use Committee. The colon cancer mid-density tissue array (purchased from US Biomax Inc. Rockville, MD, USA) and the 23 human specimens of colon cancer and normal tissues (LSUHSC pathology core) fall under IRB exemption type 4.

### Animals and treatment

WT and ICAD^−/−^ mice, both maintained on a C57BL/6×129/sv background, were bred in a specific-pathogen free facility at LSUHSC, New Orleans, LA, and allowed unlimited access to sterilized chow and water *ad libitum* and were maintained on a 12-hour light-12-hour dark schedule. At 5 weeks of age, mice (12 animals per group) received an *i.p.* injection of 20 mg/kg DMH (Sigma, St. Louis, MO) in saline containing 1 mM EDTA once a week for eight weeks. The animals were then sacrificed by CO_2_ asphyxiation 12 or 24 weeks later. For acute exposures, mice received a single *i.p.* injection of 20 mg/kg of the drug and sacrificed either 24 or 48 h later. Colons were removed and fixed with formalin. Some mice were utilized to isolate primary CECs as described below.

### Isolation of primary colon epithelial cells, immunofluorescence, treatments, measurement of cell viability and clonogenic assay

Colons were opened longitudinally and washed extensively with calcium- and magnesium-free HBSS supplemented with antibiotics. Tissue segments were incubated for 90 min at 37°C in complete DMEM medium containing 20% FBS, 2% Luria broth, glutamine, 10 mg dispase, and 0.2% collagenase I (Worthington Biochemical Corporation, Lakewood NJ) with constant gentle agitation. The digest was washed with HBSS by centrifugation and then resuspended in complete medium without enzymes. Isolated colon crypts were then plated and incubated at 37°C in 5% CO_2_ for 24 h, after which floating crypts were transferred to a fresh plate in complete medium. The efficiency of cell isolation was evaluated by the degree of purity of the crypts and the low number of dead cells. The epithelial nature of the cells was assessed by immunofluorescence with antibodies to pan-cytokeratin (Santa Cruz Biotech, Santa Cruz, CA) as described previously [23]. For the cell death assay, CECs were prepared in 24-well plates and treated with a combination of 2.5 ng/ml TNF-α, 5 µg/ml cyclohexamide, and 3 mM butyric acid (TNF+CHX+BA) or with 1 mM DMH for 24 h as described previously. Cell viability was assayed, in quadruplicate samples, using calcein-AM staining as previously described [23]. For the clonogenic assay, WT and ICAD^−/−^ mouse fibroblasts (described [13]) were exposed to IR (2 Gy), after which the cells were seeded in 35-mm dishes in 0.5% low-melting point agarose on a bed of 1% noble agar in complete DMEM medium. Colonies were scored with an automated counter and the results are expressed as fold-increase compared with the value for sham-irradiated WT cells.

#### Histopathology and Immunohistochemistry (IHC)

Serial sections from paraffin-embedded colonic tissues were prepared using standard methods. Every fifth section was subjected to H&E staining for routine histological evaluation. Pycnotic/apoptotic cells were determined by nuclear condensation, cellular fragmentation, shrinkage, and/or detachment from the mucosa, aided with TUNEL assay (Millipore, Billerica, MA). The TUNEL assay was not used as a primary means to determine pycnosis/apoptosis given that ICAD^−/−^ cells failed, in general, to display obvious positive signals upon labeling. The colon cancer mid-density tissue array (103 cases/208 cores) was purchased from US Biomax Inc. (Rockville, MD, USA). Twenty three specimens of colon cancer and adjacent normal tissues were acquired from the LSUHSC pathology core. Tissue sections were subjected to IHC with primary antibodies to ICAD (Santa Cruz Biotech, Santa Cruz, CA), poly(ADP-ribose) (BD Pharmingen, San Diego, CA), PCNA (Novusbio, Littleton, CO), MCP-1 (Santa Cruz Biotechnology, Santa Cruz, CA), COX-2 (Santa Cruz Biotechnology, Santa Cruz, CA) as described previously [24]. Assessment of immunoreactivity and comparisons between groups were conducted blindly by a certified pathologist (L. Del Valle). For tissue array analysis, ICAD immunoreactivity was classified as high (≥4+), moderate/high (3+), moderate/low (2+), low (1+), or negative (−). The results are expressed as percent positivity of total samples (cancer or normal).

#### Identification and count of ACF and tumors

ACF were identified in H&E-stained serial sections on the basis of the presence of atypical nuclei, size, increased pericryptal area, and thickened layer of epithelial cells aided by the assistance of a pathologist (*K. Fallon*). ACF with 2 or more glands were included in the assessment. Diameter of tumors was assessed using a stereomicroscope.

#### Profiling of chromosomal aberrations by aCGH

The profile of chromosome aberrations in colonic tissue (isolated from paraffin-embedded tissues) from DMH-treated WT or ICAD^−/−^ mice was assessed using an oligo microarray-based CGH with a chip containing 105,000 mouse genomic probes (Agilent, Santa Clara, CA; www.agilent.com). The reference controls were DNA isolated from colon tissue of age-matched naïve WT or ICAD^−/−^ mice. The reference and test DNAs were digested with *Alu* I and *Rsa* I (Promega, Madison, WI), and purified with the QIAprep Spin Miniprep kit (QIAGEN, Germantown, MD). Test DNA (1 µg) and reference DNA (1 µg) were labeled by random priming with Cy5-dUTP and Cy3-dUTP, respectively, using the Agilent Genomic DNA Labeling Kit Plus. The individually labeled test and reference samples were concentrated using Microcon YM-30 filters (Millipore, Billerica, MA) and then combined. Following probe denaturation and pre-annealing with mouse Cot-1 DNA, hybridization was performed at 65°C with rotation for 40 h at 20 rpm. Four steps were done with Agilent Oligo CGH washes: Wash buffer 1 at room temperature for 5 min, wash buffer 2 at 37°C for 1 min, an acetonitrile rinse at room temperature for 1 min and a 30 sec wash at room temperature in Agilent's Stabilization and Drying Solution. All slides were scanned on an Agilent DNA microarray scanner. Data including Copy Number Variations were obtained by Agilent Feature Extraction software 9 and analyzed with Agilent Genomic Workbench software 5.0, using the statistical algorithms z score and ADM-2 according to sensitivity threshold respectively at 2.5 and 6.0 and a moving average window of 0.2 Mb.

### Data analysis

All data are expressed as means ± SD of values from at least six mice per group or from 4 to 6 replicates of the same treatment of the cultured cells. PRISM software (GraphPad, San Diego, CA) was used to analyze the differences between experimental groups by one-way ANOVA followed by the Dunnett's multiple comparison test. The analysis of the aCGH data was described above.

## Results

### Association between ICAD deficiency and colon malignancy in humans

**Figure 1:**
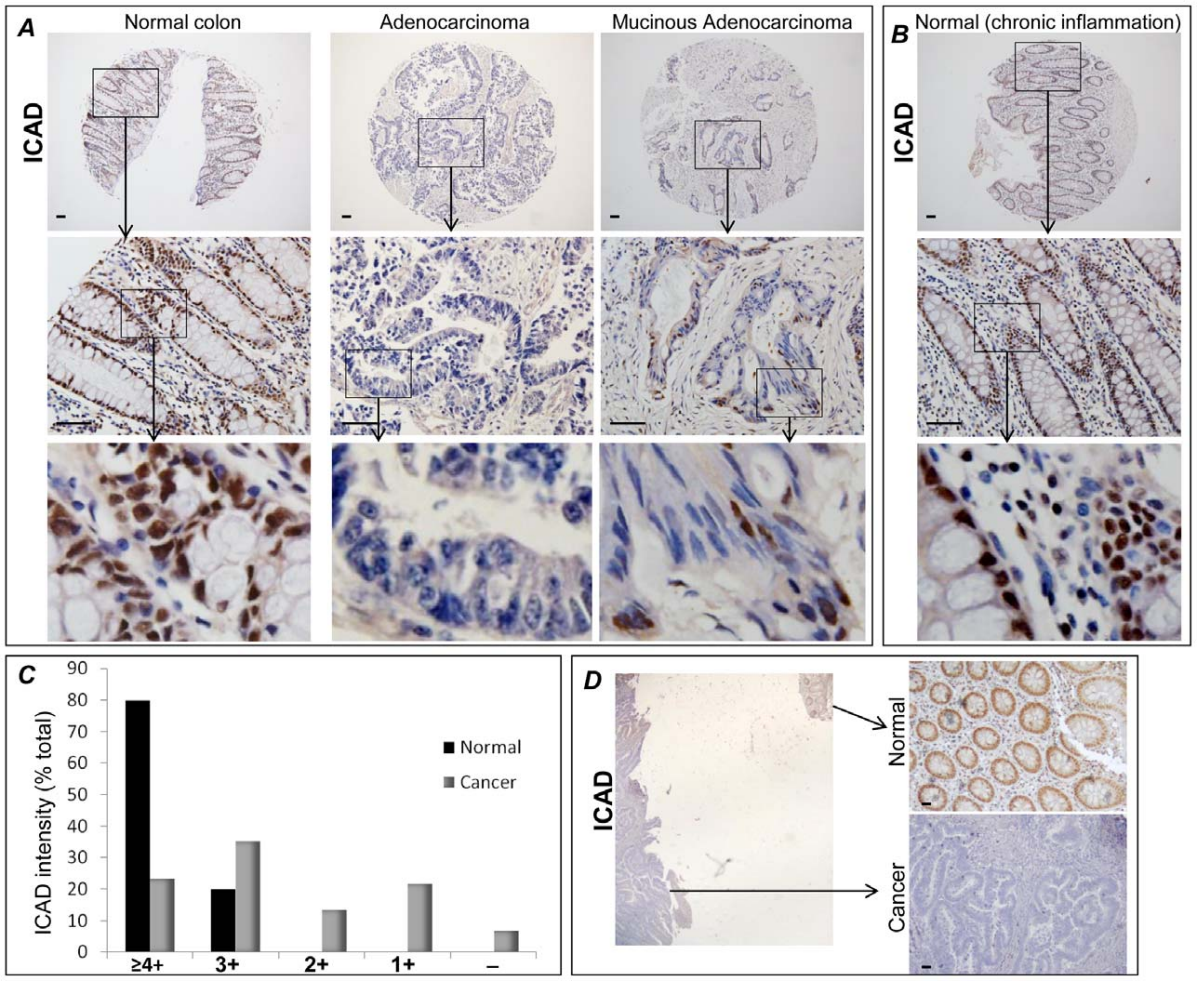
Association between ICAD deficiency and colon malignancy in humans. (**A**) Tissue arrays containing sections from normal colon (n = 60) or cancer (n = 131) tissues were subjected to immunohistochemical staining with antibodies against human ICAD. (**B**) Expression levels of ICAD in chronically inflamed colon. (**C**) The extent of ICAD immunoreactivity was assessed and classified as high (≥4+), moderate/high (3+), moderate/low (2+), low (1+), or negative (−); The results are expressed as percent positivity of total samples (cancer or normal). (**D**) Normal and cancerous tissues were fixed in formalin, embedded (both normal and diseased tissues from each patients were embedded together), sectioned, and then subjected to IHC with antibodies to human ICAD. Black boxes in (**A**, **B**, and **D**) indicate the magnified regions.

We initiated our studies by conducting a direct examination of ICAD expression using immunohistochemistry in an array of both colon cancer and normal tissues. The expression of ICAD appeared to be more prominent in epithelial cells of normal colonic mucosa with a primary nuclear subcellular localization (Fig. 1A). ICAD expression levels, however, were compromised in the cancerous tissues (Fig. 1A). It is important to note that chronic inflammation and the presumed stress responses associated with such inflammation did not appear to affect the expression levels or subcellular localization of ICAD in the colon (Fig. 1B). Figure 1C provides a quantitative assessment of ICAD expression levels in the tissue array: more than 40% of cancer tissues were considered to be markedly depleted of ICAD. It is understood that the strength of the data attained using tissue arrays may be limited given the fact that they may not include normal adjacent colonic tissues as internal controls. To circumvent this limitation we examined the expression of ICAD in 23 human specimens of colon cancer in conjunction with normal tissue collected from the same patients. Analysis of the tissues indicated that ICAD expression levels were compromised in the cancerous tissues as shown in Figure 1D (lower panel) as compared to that detected in the normal colonic tissue of the same patient; 17 out 23 specimens displayed a decrease in the level of ICAD compared to levels detected in adjacent normal tissues. The remaining specimens showed moderate to little change in ICAD immunoreactivity. Altogether, these results establish a potential connection between ICAD deficiency and colon malignancy. However, a more detailed investigation was required to understand and establish the relationship between ICAD deficiency and colon malignancy, particularly those related to examination of the involvement of the protein in cell fate and genetic alteration leading to tumorigenesis.

### Promotion of a tumorigenic phenotype by ICAD deficiency upon exposure to DNA damage induced by low-dose ionizing radiation (IR)

**Figure 2:**
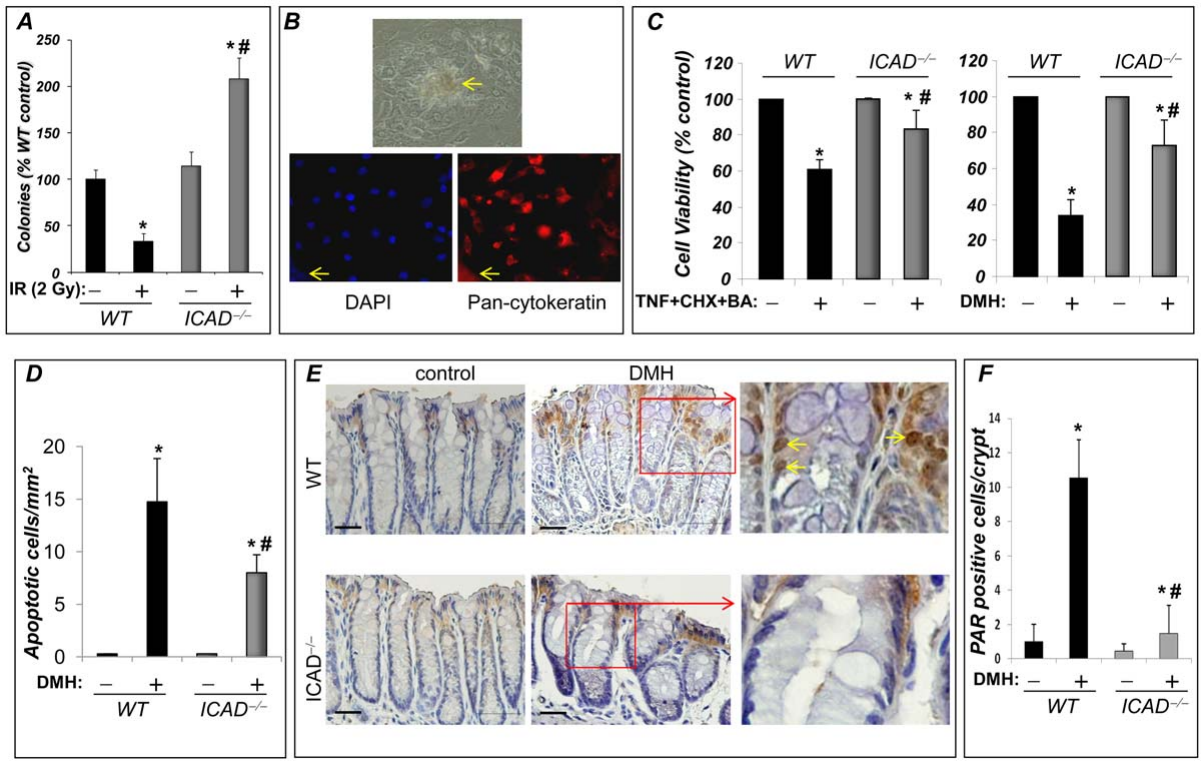
Effect of ICAD deficiency on colony formation in soft agar by fibroblasts exposed to IR and on TNF+CHX+BA or DMH-induced death in CECs. (**A**) ICAD^−/−^ and WT mouse fibroblasts were either exposed to IR (2 Gy) or sham-irradiated, after which they were cultured in soft agar with complete medium for 3 weeks. Colonies were then counted with an automated counter. Data are expressed as a percentage of the value for sham-irradiated WT cells and are means±SD of triplicates from a representative experiment. *, difference from sham-irradiated WT cells, p<0.05; #, difference from WT cells exposed to IR (2 Gy), p<0.05. (**B**) CECs were prepared from WT mice and allowed to propagate for 4 days. Cells were then fixed and subjected to immunofluorescence with antibodies to pan-cytokeratin (red) or staining with DAPI (blue); top panel is an example of CECs visualized by bright field microscopy. Yellow arrows indicate colonic crypts. (**C**) CECs, isolated from WT or ICAD^−/−^ mice, were treated with 2.5 ng/ml TNF combined with 5 µg/ml cyclohexamide and 3 mM butyric acid (designated as TNF+CHX+BA) or 1 mM DMH. Cells were incubated for 48 h in the absence or presence of TNF+CHX+BA or DMH, after which, after which cell viability was assessed by measurement of calcein-AM fluorescence. Data are expressed as a percentage of the viability of untreated cells and are means±S.D. of values from four wells from a representative experiment. *, Difference from respective control, p<0.05. #, Difference from respective treated cells, p<0.05. (**D**) Mice received an ip injection of 20 mg/kg DMH or saline (control). Mice were sacrificed after 48 h, and their distal colons were removed and formalin-fixed. Tissue sections were then prepared and subjected to H&E staining or TUNEL staining. Pycnotic/apoptotic cells were determined by nuclear condensation, cellular fragmentation, shrinkage, and/or detachment from mucosa, aided with TUNEL assay. On average, 40 crypts per section were counted. Results are expressed as the number of pycnotic/apoptotic cells per mm^2^. Data are means±SD of values from at least six mice per group. *, difference from the respective untreated mice; *p*<0.01; #, difference from WT mice exposed to DMH, *p*<0.01. (**E**) Mice were treated as in (**D**) except that they were sacrificed after 24 h. Distal colons were removed and formalin-fixed. Tissue sections were then prepared and subjected to IHC with antibodies to poly(ADP-ribose). Red boxes indicate the magnified regions; yellow arrows show poly(ADP-ribose)-immunoreactive nuclei. (**F**) A quantification of poly(ADP-ribose)-immunoreactive nuclei of CECs in the colonic mucosa of the different experimental groups.

We previously showed that ICAD deficiency reduces sensitivity to (TNF-α)-induced cell death [13], [25]. To determine whether the apoptosis-resistant phenotype of ICAD^−/−^ fibroblasts promoted their transformation after induction of DNA damage, we examined the ability of these cells to form colonies in soft agar. Wild-type (WT) and ICAD^−/−^ cells were first exposed to a low-dose (2 Gy) IR, after which they were suspended in agar with complete medium and colonies were counted after incubation for 3 weeks. Figure 2A shows that ICAD deficiency resulted in a significant (∼6.5-fold) increase in the ability of cells to form colonies in soft agar after exposure to IR; it is noteworthy that the colonies developed by wild type cells were small in number and size. These results suggest a critical role for ICAD in cell homeostasis and that ICAD deficiency may contribute to a tumorigenic phenotype upon DNA damage.

### Association between ICAD gene deletion and a decrease in sensitivity of colon epithelial cells to death stimuli

Given the potential tumorigenic effect of ICAD deficiency upon DNA damage, we examined this possibility in the colon setting *in vitro* and *in vivo*. To begin testing our hypothesis, we examined the effect of *ICAD* gene knockout on the response of primary colon epithelial cells (CECs) to inducers of cell death, for instance, TNF-α, cycloheximide, and butyric acid (TNF+CHX+BA) or to the colon carcinogen DMH. We have previously shown that the TNF+CHX+BA combination is effective in promoting cell killing in CECs [26]. Additionally, several reports by us and others showed that cyclohexamide or butyrate sensitizes a number of human colon epithelial cell lines to TNF-α-induced cell death [26], [27], [28], [29], [30]. We have recently established a method for isolating primary colon epithelial cells [26]. Figure 2B shows the typical characteristics of the epithelial cells stemming from an isolated colon crypt; the epithelial nature of the isolated cells was confirmed by immunofluorescence with antibodies against cytokeratin. Treatment of CECs with TNF+CHX+BA induced death that was moderately but significantly decreased by ICAD deficiency (Fig. 2C). Interestingly, ICAD deficiency conferred a better resistance to cell killing in response to DMH. We next examined whether ICAD deficiency also conferred resistance to DMH *in vivo* upon an acute exposure. Mice were treated with a single dose (20 mg/kg) of DMH for 24 or 48 h. Figure 2D shows that ICAD deficiency significantly reduced the number of pycnotic/apoptotic cells in colonic mucosa assessed 48 h after DMH exposure. Given our previously reported connection between ICAD expression and PARP activation [13], [16], we examined whether resistance of ICAD^−/−^ CECs to DMH-induced death was associated with a decrease in PARP activation. Figure 2E shows that DMH treatment induced a robust activation of PARP as evidenced by poly(ADP-ribose) immunoreactivity in nuclei of CECs in the colonic mucosa of treated mice. This PARP activation was largely absent in colonic epithelial cells of the mucosa of DMH-treated ICAD^−/−^ mice. A quantification of these results is depicted in Figure 2F. These results suggest that DMH-induced cell death is associated with PARP activation and that ICAD expression is required for efficient induction of such cell death. More importantly, these results suggest that the resistance to death conferred by ICAD deficiency may participate in the overall process of tumorigenesis in the colon.

### Enhancement of susceptibility to DMH-induced colon tumorigenesis by ICAD deficiency potentially post-ACF formation

**Figure 3:**
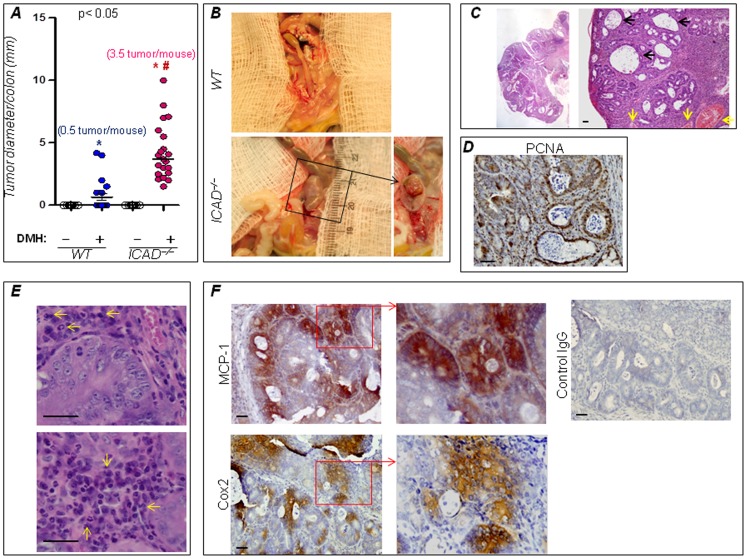
ICAD deficiency increases susceptibility to DMH-induced colon tumorigenesis. WT and ICAD^−/−^ mice received DMH injections once a week for 8 weeks. Mice were sacrificed 24 weeks later. (**A**) Diameter of tumors was assessed in the different experimental groups; n = 12. *, Difference from DMH-treated WT mice, p<0.01. (**B**) A gross examination of a large tumor detected in DMH-treated ICAD^−/−^ mice. (**C**) H&E-stained colonic tissue with a large tumor from a DMH-treated ICAD^−/−^ mouse; note the large necrotic cores (black arrows) and vascularization (yellow arrows). (**D**) PCNA expression in colon tumors from DMH-treated ICAD^−/−^ mice as assessed by IHC. (**E**) H&E-stained section with a tumor from a DMH-treated ICAD^−/−^ mouse with inflammatory cells (arrows) in the vicinity of the tumor. (**F**) MCP-1 and COX2 expression in colon tumors from DMH-treated ICAD^−/−^ mice as assessed by IHC; the right panel is an IgG control.

To further test our abovementioned hypothesis, we examined the effect of ICAD gene deletion in a model of colon tumorigenesis. WT and ICAD^−/−^ mice were injected intraperitoneally once a week for 8 weeks with 20 mg/kg DMH or vehicle (1 mM EDTA in saline). Mice were then sacrificed 12 or 24 weeks after the last injection. Figure 1A shows that 25% of the DMH-treated WT mice developed tumors displaying a moderate to marked resistance to DMH-induced tumorigenesis, a trait that is consistent with the reported resistance of the C57BL/6 and 129/Sv strains to colon tumorigenesis induced by DMH or its byproduct azoxymethane [31]. However, 58.3% of the DMH-exposed ICAD^−/−^ mice developed tumors, confirming the tumorigenic potential observed *in vitro*. Whereas the average tumor per mouse in WT mice was ∼0.5, the average of tumors in ICAD^−/−^ mice was ∼3.5 about 7-fold higher, suggesting that ICAD deficiency not only increased the susceptibility of mice to DMH but also increased tumor multiplicity. The tumors detected in DMH-treated ICAD^−/−^ mice varied in size but reached sizes as large as 1 cm as shown in Figure 3A–B. Hematoxylin and eosin staining revealed typical colon tumors with marked hyperplasia and dysplasia and development of large ACF (Fig. 3C). These tumors showed high PCNA positivity indicative of high rates of proliferation (Fig. 3D). Interestingly, these tumors exhibited large necrotic areas and displayed substantial inflammation as evidenced by the high number of inflammatory cells within the vicinity of tumors (Fig. 3E), which correlated with high MCP-1 immunoreactivity, a major chemokine in inflammatory cell recruitment, as well as expression of COX-2 (Fig. 3F).

**Figure 4:**
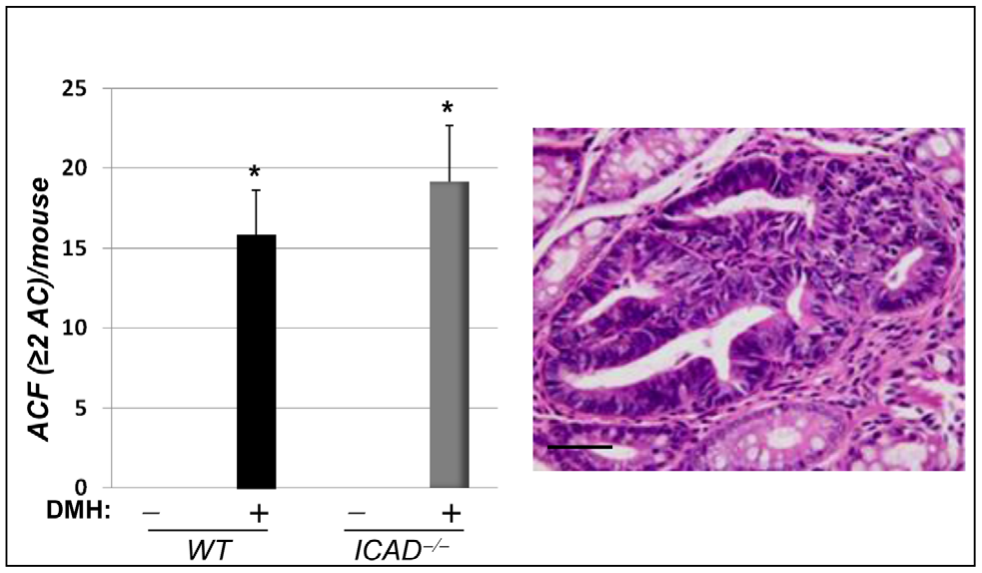
Effect of ICAD deficiency on ACF formation in DMH-treated mice. WT and ICAD^−/−^ mice received DMH injections once a week for 8 weeks. Mice were sacrificed 12 weeks later. ACF were identified and counted in H&E-stained serial sections. Only ACF with 2 or more glands were included in the assessment; n = 12. * Difference from DMH-treated WT mice, p<0.05. The right panel represents an example of a large ACF in colon of a DMH-treated ICAD^−/−^ mouse.

Genetic alterations that render CECs resistant to apoptosis or more proliferative are critical initiating events in colon tumorigenesis [1]. The subsequent accumulation of these modified epithelial cells contributes to ACF formation. These precancerous lesions may or may not progress into tumors but are generally considered to be a prerequisite for tumor progression [5]. We therefore examined the effect of ICAD deficiency on ACF at an earlier time point (12 weeks after the last DMH injection) and prior to development of large adenomas. Surprisingly, although the number of ACF in colons of DMH-treated ICAD^−/−^ mice trended higher than those detected in the WT counterpart, the difference did not reach statistical significance (Fig. 4). These results suggest that ICAD deficiency played a larger role in tumor progression rather than in initiation.

### Promotion of genomic instability by ICAD deficiency upon DMH exposure in mice and effects on colon cancer-related genes

**Figure 5:**
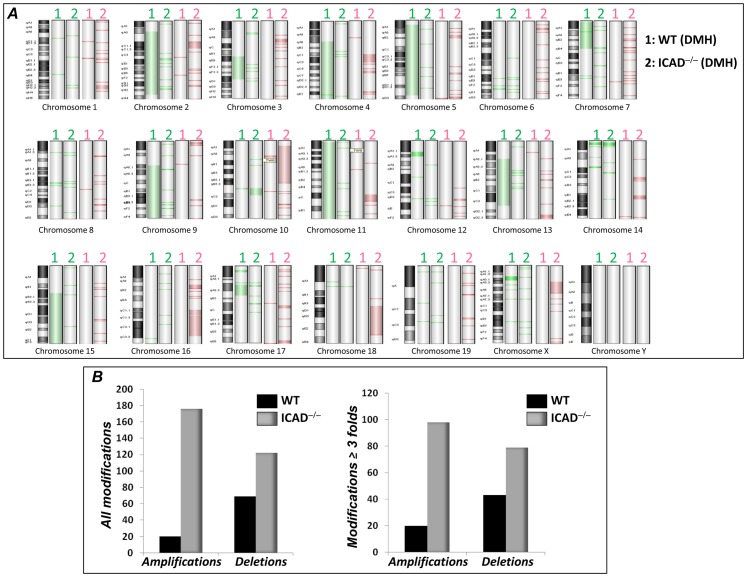
ICAD-deficiency is associated with severe genomic instability in colon of DMH-treated mice. Chromosomal DNA isolated from the different experimental groups was assessed for general chromosomal aberrations using an oligo microarray-based CGH with a chip containing 105,000 mouse genomic probes. DNA from colon tissue of age-matched naïve WT or ICAD^−/−^ mice were used as reference controls. Test and reference DNA were labeled by random priming with Cy5-dUTP and Cy3-dUTP, respectively, using the Agilent Genomic DNA Labeling Kit Plus. The individually labeled test and reference samples were concentrated and then combined. Following probe denaturation and pre-annealing with mouse Cot-1 DNA, hybridization was performed as described in the methods. Data including Copy Number Variations were obtained by Agilent Feature Extraction software 9 and analyzed with Agilent Genomic Workbench software 5.0, using the statistical algorithms z score and ADM-2 according to sensitivity threshold respectively at 2.5 and 6.0 and a moving average window of 0.2 Mb. (**A**) Depiction of amplifications (pink) and deletions (green) throughout the 19 somatic chromosomes and the X and Y chromosomes. (**B**) Quantitative assessment of total aberrations (left panel); the right panel represents data attained when stringency was increased and a cutoff of a 3-fold change was applied; p<1×E^−11^.

CAD deficiency was recently associated with genomic instability in skin carcinogenesis and in irradiated cell lines [20], [21]. Inhibition of DNA fragmentation by a caspase-3-resistant mutant of ICAD led to increased chromosome aberrations in a p53-independent manner [32]. We next wished to examine whether the increase in tumor susceptibility in DMH-treated ICAD^−/−^ mice was associated with an alteration in genomic integrity in colons of DMH-treated ICAD^−/−^ mice. To this end, we performed array comparative genomic hybridization (aCGH) analyses of tumor-containing colon tissues of ICAD^−/−^ mice and compared them with the WT counterparts. Figure 5 shows that treatment with DMH caused mild genomic instability in WT mice, which consisted primarily of deletions most prominently in chromosomes 2, 9, 11, 13, and 15. In sharp contrast, the modifications in colonic tissues of DMH-treated ICAD^−/−^ mice were extensive and consisted chiefly of amplifications. Figure 5B displays quantitative assessments of the changes exemplifying the contribution of ICAD to genomic instability. It is noteworthy that, whereas ICAD deficiency was associated with 170 amplification and 120 deletion foci, ICAD proficiency was associated with blockade of most amplifications (20 foci) with a moderate effect on deletions (68 foci). When stringency was increased and a cutoff of a 3-fold change was applied, the numbers of modified sites markedly decreased but the trend remained the same (Fig. 5D), further confirming the involvement of ICAD in genomic integrity.

Analysis of the sequences that were modified in both experimental groups shows a substantial amplification of genes necessary for cell growth or that have been associated with colon carcinogenesis. Table 1 displays several of these genes that are highly relevant to colon carcinogenesis. Notably, although the integrity of the tumor suppressor gene *p53* remained intact, a number of genes whose products are known to alter its function or associated signaling were modified. For instance, LMO3, whose gene was amplified more than 5-fold, was shown to interact with p53 and inhibit its transcriptional activity [33]. Gelsolin (*GSN*), whose gene was amplified more than 3-fold in modifications in colonic tissues of DMH-treated ICAD^−/−^ mice, was very recently shown to bind p53, leading to inhibition of p53-induced apoptosis by anchoring of p53 in the cytoplasm [34]. Additionally, whereas the *RAS* gene was not altered in DMH-treated ICAD^−/−^ mice, *RAF-1*, a critical kinase that influences RAS function with an important function in colon carcinogenesis [35] and may be associated with an increase in genomic instability [36], was amplified close to 6-fold. Frizzled homolog 6 (Fzd6), whose gene product was markedly affected by deletion (5.8-fold), functions as a negative regulator of the canonical Wnt/β-catenin signaling cascade and consequently inhibits processes that trigger oncogenic transformation, cell proliferation, and inhibition of apoptosis [37]. Interestingly, polymorphisms in *Fzd6* have been recently suggested to constitute a risk for papillary thyroid cancer with a potential involvement in maintenance of genomic integrity [38]. Altogether, the outcome of these amplifications and deletions was a significant increase in susceptibility of ICAD^−/−^ mice to DMH-induced colon tumorigenesis.

**Table 1:** Genes that were modified in colon of DMH-treated ICAD^−/−^ mice compared to untreated mice.

Gene	Chromosome location	Amplification/Deletion	P value	Function
Lmo3	6qG1	+5.139578	2.18E–24	Transcription co-factor interacts with p53 and regulates its function [33].
Raf1	6qE3	+5.632913	3.81E–29	Part of the RAS signaling pathway which promotes metastasis, angiogenesis, and loss of growth control [35]
Fzd6	15qB3.1	−5.81115	2.20E–11	Wnt receptor, functions as a negative regulator of the Wnt/beta-catenin pathway [37].
Rfwd2	1qH1	−2.75418	2.27E–11	Ubiquitin protein ligase directly interacts with p53 and JUN [50].
GSN	2qB	+3.458295	7.75E–16	bind p53, leading to inhibition of p53-induced apoptosis by anchoring of p53 in the cytoplasm [34]
Cdgap	16qB4	+4.660406	6.38E–21	Rho GTPase activating protein important for protein trafficking and cell growth and TGFβ induced breast cancer invasion [51]
Fhit	14qA1	−6.57326	1.10E–14	Tumor suppressor found in about half of all esophageal, stomach, and colon carcinomas [52].
Foxp1	6qD3	+3.803555	6.53E–10	Transcriptional factor, expression in breast cancer is associated with a worse outcome [53]
Scd1	19qC3	−7.45073	8.19E–19	Depletion of Scd1 inactivates is associated with a reduction in AKT and GSK-3 phosphorylation and impairs proliferation of cancer cells [54].

Amplifications are designated as (+) and deletions as (−).

Although the genes of critical relevance to colon carcinogenesis were not affected by DMH treatment in WT mice, the *fibroblast growth factor* gene (*fgf*)7, whose product is a member of the FGF family of proteins that signal through FGF receptors [39], was significantly amplified (4.3 folds). The *fgf7* gene remained unaltered in colonic tissues of DMH-treated ICAD^−/−^ mice. These distinct and numerous differences between the susceptibility of WT and ICAD^−/−^ mice to genomic instability may explain the different susceptibility of the two strains to colon tumorigenesis.

## Discussion

The development of resistance to apoptosis remains an important mechanism for the initiation of tumorigenesis. This resistance allows some cells to acquire new genetic modifications, which permit them to become more proliferative than normal cells. These cells will then have a growth advantage leading them to contribute to tumor progression. In this study, we have established an association between ICAD deficiency and colon malignancy in specimens from patients with colon cancer. We demonstrated, in the present work as well as in others [13], [16], that DFF is not only merely a passive factor in apoptotic fragmentation of genomic DNA but may be a factor that could influence cell fate. Additionally, DFF deficiency may render cells resistant to some apoptotic stimuli including those associated with colon homeostasis such as TNF and butyric acid, to carcinogens such as DMH and IR, or to therapeutic drugs such as etoposide. The resistance of ICAD-deficient cells to death may be associated with failure to induce PARP-1 overactivation, which is known to lead to massive depletion of intracellular NAD and collapse of mitochondria [13], [23]. We showed that when cells are damaged, for instance, in the case of exposure to low-dose IR, ICAD deficiency promoted acquisition of a tumorigenic phenotype. Using an animal model of colon cancer, we showed that ICAD deficiency increased susceptibility to DMH-induced colon tumorigenesis potentially by promoting progression of ACF into tumors after accumulation of numerous genetic alterations and genomic instability that allowed the amplification of tumor-promoting and deletion of tumor-suppressing genes. Interestingly, these tumor-promoting traits of ICAD deficiency appeared to occur even in the absence of any discernable effect on the tumor suppressor p53 gene.

ICAD expression and mutations were recently linked to some human cancers and to an animal model of skin cancer [20], [21], [32], [40], [41]. Along with these reports, our study strengthens the notion that DFF is an important factor in tissue homeostasis by participating in the maintenance of genomic stability. Li's group recently showed that CAD (DFF40) plays a significant role in regulation of horizontal DNA transfer as cells with inhibited DFF function are poor donors for horizontal gene transfer [32]. They also found that irradiation of DFF-inhibited cells leads to increased chromosome aberrations and aneuploidy when compared with their parental controls, which is consistent with our results using fibroblasts derived from ICAD^−/−^ mice. Konishi et al. [20] found that ICAD mRNA expression was significantly lower in esophageal squamous cell carcinoma tumors with higher pathologic stage, higher tumor status, lymph node metastasis, or more extensive lymphatic invasion. Patients who had low ICAD mRNA expression had a significantly shorter survival after undergoing surgery compared with patients who had high ICAD mRNA expression. Additionally, expression of ICAD as well as PARP-1 was reported to be altered in endometrial carcinomas compared with non-neoplastic endometrial tissues, indicating impaired mechanisms of apoptosis in the former [42]. Furthermore, Abel et al. [43] showed that the human *ICAD* gene was mapped to chromosome region 1p36.2, which is a region alleged to involve one or several tumor suppressor genes in neuroblastoma tumors. This group showed that three different coding alterations were found in the region encoding the ICAD N-terminal regulatory domain [21]. In contrast to our findings and the aforementioned reports, Brustmann reported that ICAD expression is frequently upregulated in ovarian serous carcinomas and may serve as a marker of aggressive behavior with prognostic value [22]. Collectively, our findings and the aforementioned reports lend strong support to the notion that DFF may play an important role in carcinogenesis in general and colon cancer in particular.

Although the resistance to cell death conferred by ICAD deficiency remains unresolved because few reports have indicated that such deficiency caused no significant changes in sensitivity of cells *in vitro* to some apoptotic stimuli [44], the overall conclusion is that the protective effect of ICAD deficiency may be stimulus and/or cell specific. Our results clearly show that ICAD^−/−^ CECs exhibited a higher resistance to DMH than to the TNF+CHX+BA combination. Additionally, ICAD^−/−^ fibroblasts show a substantial resistance to a combination of TNF and cycloheximide as shown in our previous reports [13], [16]. Furthermore, we have shown that ICAD^−/−^ cortical neurons exhibited a significant resistance to staurosporine, whereas these cells showed little or no resistance to etoposide [45]. Clearly, in the context of acute injury including traumatic brain injury or heart or brain ischemia, resistance of a large number of cells to death may be crucial for prevention of injury; however, in carcinogenesis, the number of cells needed to form a tumor may be markedly less and hence the resistance of a small number of cells to DMH, for instance, may be sufficient to contribute to tumor formation. It is interesting that the difference in ACF formation trended higher in ICAD^−/−^ mice upon repeated treatment with DMH compared with the WT counterparts; however, it was statistically insignificant. Although these results may suggest that resistance of ICAD^−/−^ CECs to DMH-induced death may not contribute to ACF formation and initiation of tumorigenesis, it is highly likely that this resistance plays an important role in progression of ACF to tumors. With the added role of DFF in genomic stability, ICAD deficiency appears to affect the number and size of tumors in DMH-treated mice. By decreasing susceptibility to apoptosis, CECs may accumulate additional mutations leading to clonal selection [4], facilitating, as a result, acquisition of a tumorigenic phenotype.

Although it is increasingly evident that DFF plays a role in genomic stability, the mechanism by which the apoptotic endonuclease complex participates in such a vital and continuous process is largely unclear. A more puzzling issue is the fact that ICAD deficiency was associated with a large number of amplifications upon DMH treatment. It is tempting to speculate that the endonuclease activity intrinsic in CAD may participate in preventing amplification of genomic DNA. However, it is widely established that the function of CAD requires caspase activity and inactivation of ICAD [46]. Accordingly, one would presume that the function of DFF in genomic stability would require caspase activation. Interestingly, caspase-3, a potent inactivator of ICAD, has been reported to function in living cells without ultimately causing death [47] and may be important to processes such as monocyte differentiation into macrophages [48]. Therefore, it is possible that CAD becomes active during normal growth of cells and participates in maintaining genomic integrity. The latter possibility is unlikely given that our examination of caspase-3 activation in normal colonic tissue by immunohistochemistry with antibodies against the active peptide of the enzyme revealed no immunoreactivity (data not shown). Similarly, caspase-3 activation was not detected in normal colon tissues of A/J or AKR/J mice [26]; the latter mouse strains are susceptible or resistant to DMH-induced colon tumorigenesis, respectively. It is then possible that DFF functions in genomic stability in a manner that is completely independent of caspase-3 activation. Obviously, more experimentation is necessary to shed light on such a mechanism. It is rather important to acknowledge that some of the complexity of the effects of ICAD deficiency in our animal model may be related to the fact that the ICAD gene is deleted in all cell types and tissues. This becomes even more relevant when one considers the potential involvement of cells other than those from the colon such as inflammatory cells, which play an important role in colon carcinogenesis. Additionally, it is noteworthy that *in vivo* compensatory mechanisms that take place in response to gene deletion may modify tissue responses to carcinogens such as DMH. An ideal approach would be to examine the relationship between ICAD deficiency and colon cancer in mice with a colon-specific ICAD gene deletion.

Our aCGH analysis revealed that a number of genes that have high relevance to overall carcinogenesis and colon cancer have been modified. It is interesting that all these modifications occurred in the absence of any detectable effects on *RAS*, *p53*, *APC*, or *β-catenin* genes. However, several genes that can affect the aforementioned factors have been altered, namely *RAF-1*, *GSN*, *LMO3*, *Fzd6*, and several others. The alteration in the latter genes may constitute the driving forces for increased susceptibility of the ICAD^−/−^ mice to tumorigenesis in response to DMH treatment. Although the confidence in the selected modifications is high given the very low p-values associated with these changes, their validation by immunohistochemistry may be necessary particularly with respect to amplifications because the levels of some gene products can be regulated at the translation level or post-translationally. Additionally, an intact p53 gene does not necessarily indicate an intact protein with a normal function given the possibility that ICAD deficiency could alter, directly or indirectly, the fate, phosphorylation, or overall function of the tumor suppressor. This is also true to the other genes including *RAS*, *APC*, and *β-catenin*. The mild genomic instability induced by DMH in wild type mice is not surprising as similar results were reported by Rosenberg's group in which the A/J mice, a strain with a normal level of wild type ICAD (data not shown), exhibited a low level of genomic instability upon exposure to azoxymethane, the byproduct of DMH [49]. It is noteworthy that the A/J mice are susceptible to DMH or its byproduct azoxymethane and develop colon tumors [49]. The role of genomic instability in such tumorigenesis appears to be minimal. Altogether, our results present a case for ICAD deficiency in colon carcinogenesis and that apoptosis and genomic instability may constitute the means by which such a deficiency may contribute to the process of increased susceptibility to carcinogen-induced tumorigenesis.
